# Exploring the relationship between Nutrition, gUT microbiota, and BRain AgINg in community-dwelling seniors: the Italian NutBrain population-based cohort study protocol

**DOI:** 10.1186/s12877-020-01652-2

**Published:** 2020-07-23

**Authors:** Federica Prinelli, Nithiya Jesuthasan, Marco Severgnini, Massimo Musicco, Fulvio Adorni, Maria Lea Correa Leite, Chiara Crespi, Sara Bernini

**Affiliations:** 1 Institute of Biomedical Technologies-National Research Council, Via Fratelli Cervi, 93 20090 Segrate, MI Italy; 2 IRCCS Mondino Foundation, Neuropsychology/Alzheimer’s Disease Assessment Unit, Via Mondino 2, 27100 Pavia, Italy; 3 Scuola Universitaria Superiore IUSS Pavia, Nets Center, Piazza della Vittoria, 15 -, 27100 Pavia, Italy

**Keywords:** Cognitive impairments, Dietary habits, Observational study, Gut microbiota, Brain measures, Gut-brain axis

## Abstract

**Background:**

Epidemiological evidence suggests that healthy diet is associated with a slowdown of cognitive decline leading to dementia, but the underlying mechanisms are still partially unexplored. Diet is the main determinant of gut microbiota composition, which in turn impacts on brain structures and functions, however to date no studies on this topic are available. The goal of the present paper is to describe the design and methodology of the NutBrain Study aimed at investigating the association of dietary habits with cognitive function and their role in modulating the gut microbiota composition, and brain measures as well.

**Methods/design:**

This is a population-based cohort study of community-dwelling adults aged 65 years or more living in Northern Milan, Italy. At the point of presentation people are screened for cognitive functions. Socio-demographic characteristics along with lifestyles and dietary habits, medical history, drugs, functional status, and anthropometric measurements are also recorded. Individuals suspected to have cognitive impairment at the screening phase undergo a clinical evaluation including a neurological examination and a Magnetic Resonance Imaging (MRI) scanning (both structural and functional). Stool and blood samples for the gut microbiota analysis and for the evaluation of putative biological markers are also collected. For each subject with a confirmed diagnosis of Mild Cognitive Impairment (MCI), two cognitively intact controls of the same sex and age are visited. We intend to enrol at least 683 individuals for the screening phase and 240 persons for the clinical assessment.

**Discussion:**

The NutBrain is an innovative study that incorporates modern and advanced technologies (i.e. microbiome and neuroimaging) into traditional epidemiologic design. The study represents a unique opportunity to address key questions about the role of modifiable risk factors on cognitive impairment, with a particular focus on dietary habits and their association with gut microbiota and markers of the brain-aging process. These findings will help to encourage and plan lifestyle interventions, for both prevention and treatment, aiming at promoting healthy cognitive ageing.

**Trial registration:**

Trial registration number NCT04461951, date of registration July 7, 2020 (retrospectively registered, ClinicalTrials.gov).

## Background

Human aging can be viewed as a complex and multifactorial phenomenon resulting from an interaction between genetic background, environmental factors, epigenetic and stochastic events [1]. Aging is the main risk factor for the development of several non-communicable diseases such as cancer, diabetes, cardiovascular and neurodegenerative diseases. Since population grows older, we expect an increase in the occurrence of these age-related diseases [2]. Among them, dementia and cognitive impairment are becoming leading causes of disability in the older population [3]. In 2015, about 47 million people were affected by dementia worldwide. In European countries, the prevalence is about 6.4% in people aged 65 years or older [4] with an incidence that doubles every 5.9 years, increasing with population age, varying from 3.1 cases per 1000 person-years in the age group 60–64 to 175 cases per 1000 years person in the age group above 95 years [5]. The transitional phase between normal physiological and pathological aging is a clinical condition called Mild Cognitive Impairment (MCI), characterized by a deficit in one or more cognitive domains (memory, visual-spatial and executive function, attention, and language) without compromising the normal daily activities [6]. The data recently published by the COSMIC International Consortium report a prevalence of MCI in subjects aged 60 or more ranging from 7 to 21% [7, 8], with an incidence that varies from 51.0 to 76.8 cases per 1000 person-years in our country [9]. Since about 15% of MCI subjects yearly converts to dementia [10], it is important to better characterize this group of people in order to intervene in a timely manner.

To date, only limited, primarily symptomatic treatment options are available and do not have proven effects on delaying disease progression. Thus, there is a growing interest in identifying non-pharmacological strategies able to prevent or delay the onset of disease [11]. Age, sex, family history of disease and genetic susceptibility (i.e. carriers of the Apolipoprotein E ε4 genotype - APOEε4) are known but not modifiable risk factors. Therefore, an intervention on modifiable risk conditions such as hypertension, diabetes, dyslipidaemia, obesity, neuropsychiatric symptoms, and poor lifestyles (unhealthy diet, low cognitive stimulation, physical inactivity, smoking habit, and limited social network) seems to be the most promising approach [12].

Accumulating epidemiological evidence suggests that intake of specific nutrients including antioxidants (vitamin C, E, A, and carotenoids), long-chain polyunsaturated fatty acids (PUFAs – n3) and B vitamins (B9, B6, B12) is associated with a slowdown of cognitive decline and a reduced risk of dementia [13]. Despite the encouraging results from observational studies, clinical trials on vitamins and supplements report conflicting results [14], mostly because of the complexity of dietary exposure. In order to take into account for the biological interactions between different components of the food “matrix”, several dietary patterns, such as the Mediterranean Diet (MD)-type pattern or other “healthy” indices [15–17], have been developed and investigated in relation to cognitive disorders. The majority of the epidemiological studies reported a reduction in the risk of cognitive impairment for increasing adherence to MD and “Prudent” pattern, characterized by foods of plant origin, fish, poultry, and whole grains. On the contrary, the “Western” pattern, based on red and processed meat, fats, refined cereals, snacks, sugars, alcohol and a reduced fibre content, is associated with an increased risk of cognitive disorders [18–21]. Currently, a few multi-domain intervention studies including dietary components (e.g. the PREDIMED [22, 23], preDIVA [24], MAPT [25], FINGER [26] and other randomized controlled trials (RCTs) [27–29]) are performed in cognitively healthy older persons or in people at risk of developing dementia [30, 31].

The biological mechanisms through which the various dietary components could exert a protective effect on the brain mainly involve processes linked to reduce oxidative stress [32], mitochondrial function, immune dysfunction, inflammatory state, and altered nutrient-sensing mechanisms [33]. However, the exact nature of these interactions and the underlying mechanisms are poorly explored and understood.

In recent decades, research has highlighted the potential role of the human gut microbiota in the regulation of the immune system, in the absorption of nutrients, as well as in the physiology of the nervous system and brain functions [34]. Moreover, alterations to the composition of the bacterial population of the human gut have been associated with various pathological conditions in the host including some neurodegenerative disorders such as Parkinson’s disease [35] and cognitive impairment [36, 37]. Diet represents one of the main determinants of gut microbiota due to its ability to modulate the microbe population composition, which in turn, impacts on the host therefore;, a dietary intervention can be considered a valid approach in the prevention and/or treatment of these diseases [38]. Within the bidirectional interactions of the gut-brain-axis, the gut microbiome communicates to the central nervous system primarily through neuroendocrine and neuro-immune signalling mechanisms and, also, via the generation of bacterial metabolites, which exert their physiologic effects both locally and systemically [39]. Despite considerable progresses, the knowledge in this area is scarce and the mechanisms underlying this relationship are still overlooked.

An innovative approach in understanding the impact of human gut microbiota on brain health, involves the use of neuroimaging, which would foster the identification of potential mediators of this relationship [40, 41]. Furthermore, recent studies have shown that brain imaging, given the precision in measuring changes in the structures and functions of the brain associated with the aging process, can be a reliable tool for exploring the relationship between diet, gut microbiota, and brain measures [42, 43]. The ground-breaking hypothesis underlying the present study is that the strict connection between diet, gut microbiota, and brain can partly explain how our dietary habits may accelerate or slow down brain aging. Bearing these considerations in mind, the NutBrain Study (Exploring the relationship between Nutrition, gUT microbiota, and BRain AgINg) aims to understand the biological mechanisms through which diet influences cognitive disorders with a special focus on the impact of nutrition on gut microbiota and brain characteristics, by applying a novel multi-level approach that integrates traditional epidemiological methods with neuroimaging and gut microbiota profiling. Aims of the NutBrain study are: i) to estimate the occurrence of MCI and other cognitive disorders in community-dwelling older people aged 65 + years; ii) to investigate the association between lifestyle habits and cognitive ageing outcomes; iii) to explore the role of diet, in modulating the gut microbiota composition, which in turn impacts on brain structures and functions as well.

The goal of the present paper is to describe the design and methodological approach of the NutBrain study protocol.

## Methods

### Study design, setting, and participants

**Fig. 1:**
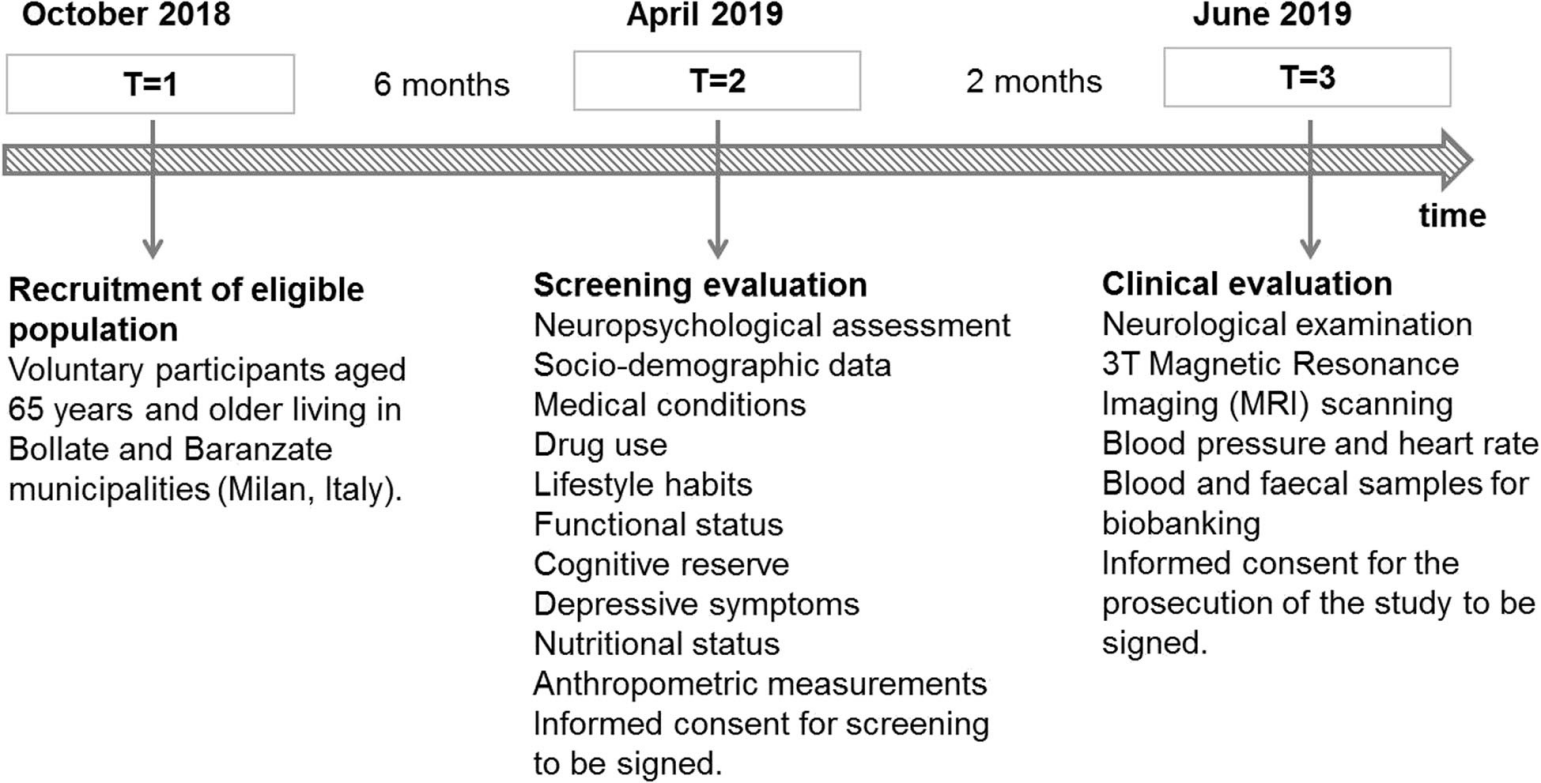
Flow-chart of the NutBrain Study design

The NutBrain Study is an ongoing population-based cohort study promoted by the Institute of Biomedical Technologies of the National Research Council (ITB-CNR, Segrate, Italy) and the Hospital IRCCS Fondazione Mondino (Pavia, Italy). The NutBrain Study is structured in three phases (Fig. 1*).*

#### T = 1 Participant’s recruitment

The study is performed in two sites in the outskirt of North-Milan: Bollate and Baranzate. A random sample is drawn from the official register of residents in the two municipalities. Inclusion criteria are: attending a medical appointment in the research facility, living at home in one of the two municipalities, and being 65+ years. Eligible population is contacted by means of a letter of invitation, in which people are invited to contact the ITB-CNR by phone to schedule the first visit during which participant is asked to bring last instrumental and clinical exams prescribed by their general practitioner. A comprehensive communication campaign is implemented to publicize and promote the project as well as to foster recruitment. To ensure a high enrolment rate, the Mayors of the two municipalities have been actively involved since the earliest planning stages of the recruitment and have granted patronage to the project. Additionally, the study is promoted and advertised at the community level through different channels: by printing brochures and setting posters in senior recreation centres, shops, pharmacies, medical doctors office, and patients associations; by articles and advertisements in local newspapers, institution websites, as well as in social media (e.g. Facebook, https://www.facebook.com/TheNutBrainStudy/) [44]. The NutBrain Study has a website (www.nutbrain.it) where potential participants are directed for further information and can register interest to participate. In addition, to raise awareness, several public events describing the objectives of the proposed study are organized for recruiting eligible people. T = 1 started in October 2018.

#### T = 2 screening evaluation

Data collection started in April 2019. Participants are visited at the research facility in their residence town (arranging home visits for disabled individuals) by a trained team. Informed consent form is completed at the research facility prior to data collection. In those individuals without capacity to give full informed consent, proxy consent is collected from relatives or caregivers. This 2-h interview includes a face-to-face administration of a neuropsychological battery of tests and questionnaires to inquire about socio-demographic, occupational, and social-economic data, education, medical conditions and drug use, lifestyle habits, functional status, and dietary behaviours. At the end of the visit, a stool sampling kit consisting of a sterile faeces container with instructions explaining the procedure for the stool sample collection, and a 3-day food diary is provided to each participant. The first visit serves to screen each participant for cognitive functions and for identifying inclusion/exclusion criteria in a more comprehensive manner.

#### T = 3 clinical evaluation

Individuals suspected to have cognitive impairment at the screening neuropsychological tests undergo a clinical examination. Participants are transported from their own home to the Hospital using a private transport service, whose cost is covered entirely by the study. The hospital visit includes a standard neurological examination performed by a neurologist and a 3 T Magnetic Resonance Imaging (MRI) scanning. At this stage, blood and stool samples are collected from each participant and stored in the biorepository at the Hospital until processing. For each subject with a clinical diagnosis of MCI two cognitively intact controls of the same sex and of the closest date of birth are enrolled and visited at the Hospital and undergoing the same protocol as MCI individuals. T = started in June 2019.

### Ethics approval and consent to participate

The study protocol and paperwork have been reviewed and approved by the Medical Ethical Committee of Pavia, Italy (Ref. number: 20180036036, 2018/04/20 and amendment Ref. Number: 20190045757, 2019/05/21). All participants provide a formal written informed consent in order to participate in the study according to the Declaration of Helsinki. In those individuals found to be without capacity to give full written informed consent, a caregiver or guardian is identified and their advice sought regarding participation.

### Data collection

A comprehensive protocol has been designed for this study. Table 1 gives an overview of the variables that are collected in the NutBrain project and the corresponding tools used for assessment.

**Table 1:** Summary of assessments and testing instruments used in the NutBrain Study

Variables	Instruments/assessment tools
**Questionnaires**	
Health and socio-demographics	Written questionnaire developed by the researchers regarding: socio-demographic information, household income, family history of diseases, medical conditions, surgery and illnesses, use of drugs and supplements, hospitalization, menstrual gynaecological history, and smoking habit.
Physical Activity level	International Physical Activity Questionnaire (IPAQ) [45]
Cognitive reserve	Cognitive Reserve Index questionnaire (CRIq) [46]
Functional status	Instrumental Activities of Daily Living scale (IADL) [47]
	Katz Index of Independence in Activities of Daily Living (ADL) [48]
Depression	Center for Epidemiologic Studies Depression (CES-D) scale
**Anthropometry**	
Height	Portable wall-mounting stadiometer SECA 213
Weight	Homologated electronic scale Tanita SC240MA
Waist and mid-upper arm circumferences	Flexible graduated measuring tape SECA 201
Fat mass (FM%) and Total Body Water (TBW%)	Bioelectrical impedance analysis (BIA) (Tanita SC240MA)
**Biological samples**	
Blood	Cells, plasma and genomic DNA (for APOE genotyping)
Stool	Alpha- and beta-diversity measures, bacterial relative abundances (indexes estimated on the 16S rRNA-based sequencing data)
**Clinical**	
Blood pressure	Sphygmomanometer
Heart rate	Heart rate monitor
**Neuroimaging**	
MRI scans	Siemens MAGNETOM 3 T scanner
**Nutritional status**	
Dietary habits	Semi-quantitative food frequency questionnaire (SFFQ) [49]
	Estimated 3-day food diary
Malnutrition	Mini-nutritional assessment (MNA) [50]
**Neuropsychological assessment**	
Global cognitive function:	MMSE [51]
Cognitive domains	
*Memory*	Free and Cues Selective Reminding Test (FCSRT) [52]
	Logical Memory test – Babcock Test [53]
	Rey-Osterrieth Complex Figure Test (ROCF) – delay recall [54]
*Execute function*	Frontal Assessment Battery (FAB) [55]
	Phonemic and semantic verbal fluency [56]
	Trial Making Test (TMT A and B) [57]
*Language*	Picture Naming Test [58]
*Visuo-spatial abilitities*	Rey-Osterrieth Complex Figure Test (ROCF) – copy [54]

#### Socio-demographics, medical, and lifestyle data

At T = 2, participants are asked about their socio-demographic characteristics including education, marital status, work history and retirement, as well as living situation (i.e. co-habitants). Information regarding the house property and the number of rooms are used as an indicator of the household income. Participants also provide information about all current and past medical conditions, surgery and illnesses, use of medications, supplements, and hospitalizations. A detailed menstrual gynecological history is obtained from female participants (age at menarche and menopause, birth-control pill and hormones replacement therapy use, and number of births and breastfeeding). Information regarding the medical history of the participant’s first degree relatives is also collected. A series of questions are used to assess type and frequency of smoking habit. Assessment of physical activity is performed using the validated International Physical Activity Questionnaire (IPAQ) [45], which includes 7 items, divided into frequency, intensity, and duration of physical activity at low (walking), moderate, and vigorous levels, in addition to total physical activity during the last week. It also includes an item about sitting time, expressed as minutes per day, measuring sedentary lifestyle on weekdays and weekend days. Functional evaluations of activities of daily living are assessed using the Katz Index of Independence in Activities of Daily Living (ADL) [48]. The index ranks adequacy of performance in the six functions of bathing, dressing, toileting, transferring, continence, and feeding. Participants are scored yes/no for independence in each of the six functions. The participants’ ability to use the telephone and transportation, medication management and handling of finances independently is assessed using the Instrumental Activities of Daily Living scale (IADL) [47]. Participation in intellectual, social, physical and leisure activities is measured using the Cognitive Reserve Index questionnaire (CRIq), which includes 20 items grouped into 3 sections: education, working activity, and leisure time, each returning a sub-score [46]. Center for Epidemiologic Studies Depression (CES-D) scale is used to measure symptoms associated with depression experienced in the last week [59].

#### Anthropometric and clinical measurements

A trained dietician measures participants’ body weight, height, fat mass, total body water, and waist and mid-upper arm circumferences during the screening evaluation. Body weight (in Kg to the closest 0.5 kg) and body composition (Fat mass [FM%] and Total Body Water [TBW%]) are assessed by bioelectrical impedance analysis (BIA) measured using a homologated leveled-platform electronic scale (Tanita SC-240MA), with participants wearing light clothing and no shoes. Height (in cm to the closest 0.5 cm) is measured with a portable wall-mounting system with the participants shoeless in the standing position (SECA 213). Body Mass Index (BMI) is calculated by the standard formula (weight/height^2^). Waist circumference and mid-upper arm circumference are measured in the middle between the 12th rib and the iliac crest, and the midpoint between the tip of the shoulder and the tip of the elbow, respectively (in cm closest to the 0.5 cm). Body circumferences are measured using a flexible graduated measuring tape (SECA 201) with the participant in the standing position. During the clinical evaluation, a nurse measures systolic and diastolic blood pressure and heart rate with the participants in the sitting, resting position using a sphygmomanometer and a heart rate monitor, respectively.

#### Dietary assessment

A trained dietician performs a detailed nutritional assessment. A semi-quantitative food frequency questionnaire (SFFQ) (adapted from the validated Willet’s questionnaire) [49] is used to collect dietary habits over the prior year. Participants are asked to indicate how often they consume a standard portion of a given item according to a nine-category frequency scale: never/seldom (lessthan once per month), 1–3 times per month, 3 times per week, 2–4 times a week, 5–6 times a week, once a day, 2–3 times a day, 4–5 times per day, and 6+ times a day. Color pictures of serving sizes of each food item are showed to help in the understanding of standard portion sizes. Participants enrolled in the third phase of the study (T = 3) are asked to keep a food diary to record a detailed description of types and amounts of food and beverages consumed over a period of 3 days before the fecal sample collection including two weekdays and one weekend day. To convert frequency data into daily energy and macro- and micronutrient intake, frequencies are multiplied by standard serving sizes using the Italian Food Composition Databases for Epidemiological studies in Italy (http://www.bda-ieo.it/) and the MetaDieta software (Meteda s.r.l., Ascoli Piceno, 227 Italy). The Mini-Nutritional Assessment (MNA) is administered as a screening tool to identify malnourished individuals or those at risk of malnutrition [60].

#### Neuropsychological assessment

**Fig. 2:**
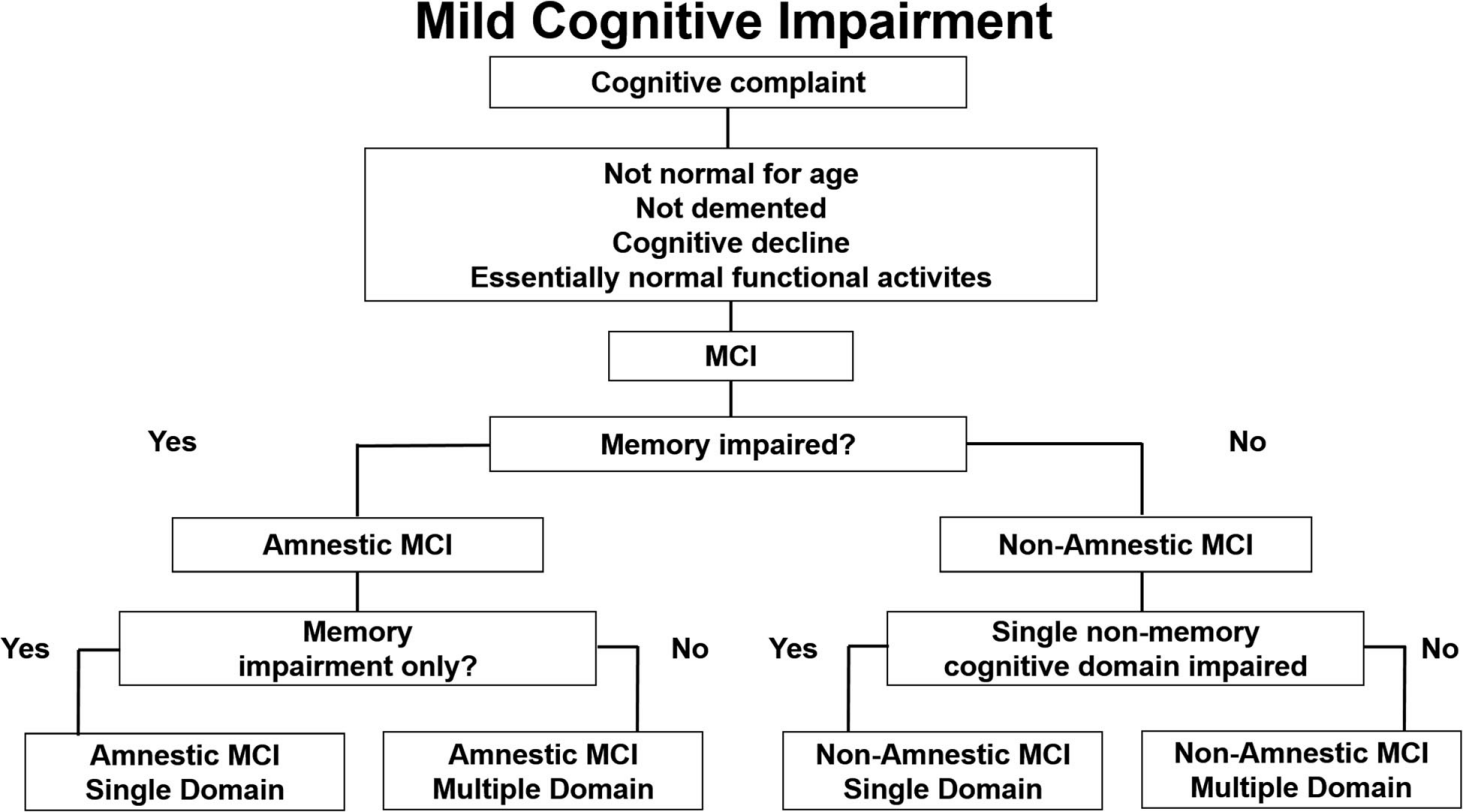
Algorithm used to classify the subtypes of Mild Cognitive Impairment (MCI) (Source: Petersen R [51].

The neuropsychological tests have been selected according to the guidelines for the diagnosis of MCI [50]. Current conceptualizations of MCI recognize multiple subtypes centered on the presence or absence of memory impairment, namely amnestic (aMCI) and non-amnestic (naMCI) and on the number of compromised cognitive domains (MCI single domain vs MCI multiple domain) (Fig. 2) [61]*.* To this scope, an experienced neuropsychologist administers the following battery of well-established neuropsychological tests exploring global cognitive function (Mini Mental State Examination-MMSE) [51] and different cognitive domains: memory (Free and Cues Selective Reminding Test (FCSRT) [52], Logical memory test - Babcock Test [53], the Rey-Osterrieth Complex Figure Test (ROCF) – delay recall [54], executive function (Frontal Assessment Battery (FAB) [55], phonemic and semantic verbal fluency [56], Trial Making Test (TMT) [57], language (Picture Naming Test [58]), visuo-spatial abilities (Rey-Osterrieth Complex Figure Test (ROCF) – copy [54]). All the test scores are corrected for age, sex, and education and compared with the values available for the Italian population (see Supplementary material).

#### Inclusion/exclusion criteria

Screened participants are selected among all subjects willing and able to undergo all test procedures including neuroimaging, blood and stool sample collection. General inclusion/exclusion criteria are the following:

*i) neuropsychological criteria:* we enrol subject with different subtypes of MCI (aMCI, naMCI, single-domain MCI, multiple-domain MCI) according to Albert’s criteria [50] and subject with normal cognitive profile. Subject with dementia, pre-existing cognitive impairment (e.g. aphasia, neglect), concomitant severe psychiatric disease, others neurological conditions (e.g. severe depression and behavioral disorders) or severe sensory disturbances (e.g. auditory and/or visual loss) that do not allow completing neuropsychological assessment are excluded from the third phase of the protocol.

*ii) microbiota’ analysis protocol*: we exclude individuals with artificial nutrition in progress; those with a history of active uncontrolled gastrointestinal disorders or diseases, including inflammatory bowel disease, ulcerative colitis, and Crohn’s disease; subjects who underwent previous major surgery on the gastro-enteric tract, with the exception of cholecystectomy and appendectomy, in the past five years, use of antibiotics or large doses of commercial probiotics in the 4 weeks prior the visit, and subjects under radio-chemo-therapy.

*iii) MRI scanning protocol*: we exclude those with metal fragments in the body, surgically implanted devices containing metal, severe claustrophobia, or inability to lie down in the MRI scanner for the duration of the study.

#### Endpoint determination

Once the presence and type of MCI have been defined by neuropsychological assessment, a certified neurologist performs the neurological assessment in order to recognize the possible cause of the clinical syndrome through a careful anamnestic investigation. To meet the core clinical criteria for MCI, it is necessary to rule out other systemic or brain diseases that could account for the decline in cognition (e.g., vascular, traumatic, medication side effects). The goal of such an evaluation is to increase the likelihood that the underlying disease is a neurodegenerative disorder. A final diagnosis is assigned by the neurologist after reaching an agreement between the neuropsychological and clinical examinations.

#### MRI measures

Neuroimaging data are acquired, pre-processed and analyzed at the Hospital. MRI data are acquired using a 3 Tesla MAGNETOM scanner (Siemens, Erlangen, Germany). MRI measures include structural (high resolution T1 anatomical scan - grey matter volume/density, cortical thickness; Diffusion Tensor Imaging scan: white matter microstructural integrity) and functional characteristics (resting state fMRI sequence: functional connectivity at rest). Preprocessing and voxelwise analysis of grey and white matter characteristics, as well as functional connectivity analysis, are carried out using appropriate toolboxes. We perform: i) standard univariate (hypothesis-driven) analyses of grey and white matter features (e.g., voxel-based morphometry, cortical thickness, tract-based spatial statistics) to obtain macrostructural and microstructural metrics discriminating patients from controls; ii) multivariate data-driven analyses of neurofunctional features (i.e., functional connectivity) and multi-modal data (i.e., integration of structural and functional features) to investigate group differences at a network level; and iii) network analysis to identify distinctive characteristics of the structural and functional connectomes in patients and controls. MRI scans are labeled according to each site’s imaging capabilities using code subject identifiers. All scans undergo a de-identification process to ensure that no subject’s identification is present in the images files.

#### Biological samples for biobanking

*Blood sample*. A qualified nurse draws 30 ml of blood sample (non-fasting state). Blood (cells and plasma) and genomic DNA (for APOE genotyping) are collected using EDTA tubes and are stored at − 80 °C in the Hospital’s biorepository according to international consensus standard operation procedures. Samples will be stored for 25 years for potential future analysis. Polymerase Chain Reaction (PCR) is performed as described by Wenham et al. [62] and the final products are digested with restriction enzyme for assessing the APOE polymorphic sites.

*Stool sample*. Participants are asked to provide a stool sample (about 250 mg) that is immediately frozen at − 20 °C and, later, stored at − 80 °C in the biorepository until processing. Total bacterial DNA is extracted using the QIAamp DNA Stool Mini Kit (QIAGEN, Valencia, CA, USA) with a modified protocol [63] based on bead-beating with glass and zirconia beads in a FastPrep-24 Instrument (MP Biomedicals, Santa Ana, CA, USA). DNA concentration and quality for each sample is determined using a NanoDrop ND-1000 spectrophotometer (NanoDrop Technologies, Wilmington, DE, USA). For each faecal sample, the V3-V4 regions of the microbial 16S rRNA gene are PCR-amplified using primers carrying specific overhang adapters appended to the primer pair sequences for compatibility with Illumina index and sequencing adapters. Taxonomy analysis are performed within the QIIME suite [64] grouping reads into Operational Taxonomic Units (OTUs) at 97% similarity and classifying them against the Greengenes database (ftp://greengenes.microbio.me/greengenes_release/gg_13_8_otus) through RDP classifier [65] Alpha-diversity (i.e.: species diversity within samples) is evaluated calculating Chao1, Shannon’s index, total number of OTUs and Faith’s phylogenetic diversity metrics, whereas beta-diversity (i.e.: diversity in bacterial composition among the samples) is estimated through weighted and unweighted Unifrac distances [66] Bacterial composition of the samples in terms of relative abundance is compared at all phylogenetic levels down to genus and differences are evaluated by non-parametric Mann-Whitney U-test for equal medians. Statistical evaluation among alpha-diversity indices is performed by a non-parametric Monte Carlo-based test, using random permutations. “adonis” function of the R package “vegan” (https://cran.r-project.org/web/packages/vegan/index.html) with random permutations is employed to determine statistical separation of the microbiota profiles.

#### Data management and confidentiality

Data are handled, monitored, computerised, and stored in accordance with the European General Data Protection Regulation (EU) 2016/679 (GDPR) (https://gdpr-info.eu/). All study records, including the consent forms, are kept in a locked filing cabinet at the ITB-CNR where the file server for data storage is located. The file server is firewalled within the ITB-CNR intranet. For privacy and security, a password granted only to the server administrator is required to access to the database. The data forms are double checked for missing data and inconsistencies. Quality of the database entered data is monitored by checking entry for a random sample of participants. Data transfer is protected by means of crypting/decrypting policy and password protection. In the final dataset a unique key identifies each subject to guarantee anonymity. Personal data are regarded as strictly confidential and removed before the exportation procedure. Security of data is guarantee via automatic backups.

#### Sample size

Given the observational nature and the “exploratory” objectives of the study design, and the large number of factors to be analyzed, formal sample size calculations for a specific aim are not feasible. However, considering an expected prevalence of individuals with MCI of approximately 20% in the general population aged over 65 from this geographical area [67] (population of resident seniors ~ 9000), a desired precision of 3% and a confidence levels of 95%, a sample size of 683 is required for the screening phase (T = 2). Conservatively assuming a maximum loss of 40% of participants to T = 3 due to ineligibility or refusal, we expect about 80 MCI individuals undergo phase 3. For each diagnosed MCI case, two cognitively intact controls (1:2 ratio, *N* = 160) of the same sex and closest in birth date to the case (±1 year) are selected from the population under investigation reaching a total of 240 individuals undergoing T = 3.

#### Planned statistical analysis

For the descriptive analysis of the variables under study, frequencies and proportions are used for qualitative variables, and mean ± standard deviations, medians ± interquartile intervals are used for quantitative variables. Subsequently, the differences between subjects with MCI diagnosis and cognitively intact controls are analyzed with respect to various variables of interest (e.g. dietary exposures, brain measures, gut microbiota composition, etc.). Comparisons between continuous variables are carried out using ANOVA tests for independent samples (for normally distributed variables) or non-parametric Mann-Whitney and Kruskall-Wallis tests (for non-normally distributed variables). Fisher exact test and χ^2^ test is used to compare categorical variables. Data are analyzed using several methodological approaches such as data reduction techniques (e.g. clusters or principal component analysis) and Compositional data analysis (CoDa) [68, 69]. Subsequently, the analysis can be extended by applying different multivariate regression models (linear and conditioned logistic) to study the relationship between the exposures and the different outcomes of interest, both in univariate and multivariate analysis by adjusting for possible confounders (e.g. socio-demographic variables, other lifestyles, etc.). Test for interaction and stratified analyzes are also performed (e.g. sex, age, or APOE genotype) to assess effect modification. The robustness of the study results are verified by conducting different sensitivity analyzes. The level of significance is set at α = 0.05 and all tests are two-tailed. Statistical analyzes are performed using the STATA software packages (version 15, StataCorp LP, College station, Texas, USA) and SPSS (IBM Corp. Released, IBM SPSS Statistics version 25.0 Armonk, NY: IBM Corp.).

#### Dissemination and provision of results to participants

Dissemination of the scientific data obtained from this project is performed through a detailed dissemination plan that takes into account for the target audience. The results of this study are communicated through peer-reviewed journals, national and international congress presentations, academic lessons, workshops, technical journals (for researchers and general practitioners), institutional and private web sites; and disseminated through associations and local printed media. On completion of the study, participants will receive a report summarizing the main results of the NutBrain Study.

## Discussion

In this study we apply an innovative “system epidemiology” [70] approach that integrates traditional epidemiological methods with modern and advanced technologies to provide new insights into the biological mechanisms underlying the relationship between dietary habits and brain aging.

The results of the NutBrain Study may have public health implications at different levels: i) unravelling the mechanisms underlying relationship between diet, gut microbiota, and age-related disorders, ii) improving the scientific knowledge with the aim to identify the best suited population for early and tailored intervention strategies, iii) providing strong evidence-based recommendations and guidelines for promoting a healthy lifestyle in target population, iv) paving the way towards new prospects for precision nutrition and medicine.

From a methodological point of view, due to the observational nature of the study design is difficult to derive causal relationship. Furthermore, our study is prone to certain biases such as reverse causation and selection bias (volunteer bias). Accordingly, the strength of the associations might be underestimated and generalization of our findings to other populations should be done with caution. However, the availability of a well-characterized cohort of elderly from the general population that includes dietary assessment, neuropsychological tests and biological samples collection, together with the integration of advanced techniques (microbiomics and neuroimaging), is a novelty and the main strength of this study. Additionally, the baseline assessment allows setting up future prospective investigation on the potential epidemiological, clinical, and biological determinants of disease progression. Because the pathological process of dementia precedes by decades the severe clinical manifestations of disease, an early intervention in asymptomatic or early mild symptomatic individuals is a promising and cost-effective solution in the agenda priorities of policymakers and governments.

In conclusion, to lessen the burden of age-related diseases and ameliorate quality of life among older people, a better understanding of the intricate mechanistic pathways subjacent brain aging is an urgent need. This “pioneer” study represents a unique opportunity to improve this knowledge, to translate into practice and to devise interventions for both prevention and treatment of age-related cognitive disorders.

## Supplementary information

**Additional file 1.** Neuropsychological assessment.

## Data Availability

Data sharing is not applicable to this article as no datasets were generated or analysed during the current study.

## Abbreviations

MCI: Mild Cognitive Impairment
MRI: Magnetic Resonance Imaging
APOE ε4: Apolipoprotein E ε4 genotype
PUFAs: Polyunsaturated fatty acids
MD: Mediterranean Diet
RCTs: Randomized Controlled Trials
IPAQ: International Physical Activity Questionnaire
ADL: Index of Independence in Activities of Daily Living
IADL: Instrumental Activities of Daily Living scale
CRIq: Cognitive Reserve Index Questionnaire
CES-D: Center for Epidemiologic Studies Depression
FM: Fat mass
TBW: Total Body Water
BIA: Bioelectrical Impedance Analysis
BMI: Body Mass Index
SFFQ: Semi-quantitative Food Frequency Questionnaire
MNA: Mini Nutritional Assessment
MMSE: Mini Mental State Examination
FCSRT: Free and Cues Selective Reminding Test
ROCF: Rey-Osterrieth Complex Figure Test
FAB: Frontal Assessment Battery
TMT: Trial Making Test
OTUs: Operational Taxonomic Units
CoDa: Compositional Data Analysis
