# Supplementary material for: Exploring the relationship between Nutrition, gUT microbiota, and BRain AgINg in community-dwelling seniors: the Italian NutBrain population-based cohort study protocol
Source: BMC Geriatr. 2020 Jul 23;20:253. doi: 10.1186/s12877-020-01652-2 (PMC7376643; doi:10.1186/s12877-020-01652-2)
Supplement: Supplementary file 1 — Additional file 1. Neuropsychological assessment. [file 12877_2020_1652_MOESM1_ESM.docx]

**Supplementary material: neuropsychological assessment**

A comprehensive battery of neuropsychological tasks was chosen to assess performance in several cognitive domains:

-The Mini Mental State Examination (MMSE) that provides a quick and easy global measure of

cognitive status in elderly people who are at high risk to develop dementia. The total score ranges from 0 to 30 with higher score indicating better global cognition;

-The Mini Mental State Examination (MMSE) that provides a quick and easy global measure of

cognitive status in elderly people who are at high risk to develop dementia. The total score ranges from 0 to 30 with higher score indicating better global cognition [54];

-The Free and Cues Selective Reminding Test (FCSRT) measures verbal learning processes, through the use of a list-learning procedure over multiple trials, and memory recall ability, semantically cued or not. This test produces different scores that allow distinguishing between long-term storage, retrieval from long-term storage and recall from short-term storage [55];

-The Logical memory test - Babcock Test, which represents a useful tool to evaluate verbal memory learning ability (encoding, storage, and retrieval processes of the memory system) as well as how the words or meaning of sentences affect the memory system. In logical memory tests, the participants are asked to memorize a short story once and then to complete an immediate recall test and 20 minutes delayed recall test [56];

-The Rey-Osterrieth Complex Figure Test (ROCF) – copy and delay recall evaluating visuospatial constructional ability, visual memory, working memory. It is also employed as a tool for measuring executive functions as planning abilities. Subjects are asked to reproduce a complicated line drawing, first by freehand copying (copy phase), and then drawing from memory (delay recall phase) [57].

-The Frontal Assessment Battery (FAB) is a behavioural battery composed of six subtests useful for the assessment of different aspects of frontal/executive functions (conceptualization, mental flexibility. motor programming, sensitivity to interference, inhibitory control, and environmental autonomy) [58];

-The Trial Making Test (TMT) for measuring visual attention, task switching, and executive function. It consists of two parts in which the subject is instructed to connect a set of 25 dots as quickly as possible while still maintaining accuracy. In Part A, patients have to connect the numbers in ascending order, with the aim to assess visual speed. Part B is similar to Part A but with the added task of alternating between numbers and letters, and it is designed to provide an expanded evaluation of frontal lobe functioning through the inclusion of tasks that assess inhibition and set-shifting ability [60];

-The Phonemic and semantic verbal fluency measures mainly executive functioning as well as some linguistic skills (such as vocabulary, articulation speed), because a successful retrieval requires executive control over cognitive process such as selective attention, mental set shifting and self-monitoring. Subjects are asked to retrieve words that start with a specific letter (e.g., F, A, S: phonemic fluency) or words that belong to a semantic category (e.g., animals, fruits, car brands: semantic fluency), typically over a 1-min period. Moreover, semantic fluency is a widely accepted measure both of executive function and access to semantic memory [59];

-The Picture Naming Test, for evaluating language abilities and in particular the capacity to access to the semantic knowledge and lexicon for visual pathway. Subjects are asked to name pictures that represent objects or living beings with high or low frequency of use [61].
